# Change in hypnotic sedative choice over time as a surrogate marker of improved performance

**DOI:** 10.1186/cc9777

**Published:** 2011-03-11

**Authors:** T Hughes, F Hanks, P Hopkins

**Affiliations:** 1 Kings College Hospital, London, UK

## Introduction

Daily sedation holds, particularly when combined with protocolised spontaneous breathing trials, are one of the only strategies available to intensivists that produce an outcome benefit [1]. This evidence has also provoked a renewed interest in the choice of both hypnotic and analgesic agents. Midazolam is known to produce unpredictable awakening and may prolong time to extubation when infusions continue longer than 48 to 72 hours. In contrast, propofol may enhance the benefit to critically ill patients of the daily sedation hold due to its pharmacokinetic properties [2]. This study examines the hypothesis that the ratio of propofol/midazolam use can be used as a surrogate marker of good practice and utilises the potential of the pharmacy procurement database.

## Methods

The amount of propofol and midazolam supplied in grams per month was obtained from the pharmacy database for both the surgical and medical critical care units for the period April 2006 to July 2009. These data were compared with the number of monthly admissions, average monthly length of stay, APACHE II score (May 2008 to July 2009) and standardised mortality rate (SMR) for that period. Sigmaplot 11.0 was used to determine statistical significance.

## Results

There was a statistically significant increase in propofol use per patient (*r *= 0.512; *P *= 0.0007) and reduction in midazolam use per patient (*r *= -0.384; *P *= 0.014) between April 2006 and July 2009. The mean ± SD monthly admission rate was 142 ± 15.3 patients. The use of propofol/midazolam was independent from length of stay and APACHE II score. Statistical significance was not reached when correlating propofol/midazolam use to fall in SMR (1.11 to 0.77) due to the limited number of data points.

## Conclusions

Although a clear relationship between reduced midazolam use and improved outcome could not be demonstrated, information from the pharmacy database remains an important means to review prescribing practice. Monthly supply may not always accurately reflect use but over time will indicate significant changes in practice such as the reduced use of midazolam at this institution.
